# Impact of Body Mass Index on Oncological Outcomes of Prostate Cancer Patients after Radical Prostatectomy

**DOI:** 10.1038/s41598-018-30473-y

**Published:** 2018-08-10

**Authors:** Young Dong Yu, Seok-Soo Byun, Sang Eun Lee, Sung Kyu Hong

**Affiliations:** 1 Department of Urology, Seoul National University Bundang Hospital, Seongnam, Korea; 2 Department of Urology, Seoul National University College of Medicine, Seoul, Korea

## Abstract

Obesity, often represented by higher body mass index (BMI), is not yet fully understood as a potential risk factor for poor clinical outcomes of prostate cancer (PCa) after radical prostatectomy (RP). This study aimed to evaluate the relationship between BMI and biochemical recurrence (BCR)-free survival in RP patients. This study retrospectively reviewed a total of 2.997 PCa patients who underwent RP between 2006 and 2017. The patients were stratified into three BMI groups according to the WHO recommendations for Asian men: normal weight (<23 kg/m^2^), overweight (≥23 to <27.5 kg/m^2^) and obese (≥27.5 kg/m^2^). Multivariable logistic regression analyses were undertaken to evaluate the factors influencing the BCR rates including BMI. Multivariable Cox regression analyses and Kaplan-Meier analyses were performed to test the association of obesity with BCR-free survival. The final pathologic results showed obese patients had greater positive surgical margin rates (13.9%, *p* < 0.001), extraprostatic invasion (19.9%, *p* < 0.001), advanced pathological Gleason score (GS) ≥ 8 (50.8%, *p* = 0.017), and lymph node invasion (LNI) (14.5%, *p* = 0.021) than overweight and normal weight patients. According to Kaplan-Meier analyses, obese patients, especially with BMI ≥ 27.5, were more likely to have lower BCR-free-survival. Multivariate Cox analysis revealed that diabetes mellitus, LNI status, pT, pathologic GS, extraprostatic invasion, margin positivity and obesity with BMI ≥ 27.5 kg/m^2^ were significantly associated with BCR-free survival after RP. Obesity (higher BMI) was significantly associated with BCR after RP. BMI ≥ 27.5 kg/m^2^ was an independent predictor of BCR-free survival.

## Introduction

As the prevalence of obesity has increased rapidly during the past two decades, its potential impact on the mortality risk of certain cancers including prostate cancer (PCa) has been emphasized^1,2^. Moreover, PCa has become a major clinical issue in many countries. Approximately 40% of PCa patients have radical prostatectomy (RP) for their definitive treatment as the current treatment guidelines for organ-confined PCa suggest definitive therapeutic modalities including RP or radiation therapy (RT)^3^. Obesity as a potential risk factor for poor clinical outcomes of PCa after RP is not yet fully understood, but several explanations have been proposed and the microenvironmental changes in obese patients is one of them. Microenvironmental changes in obese patients may increase the incidence of PCa and produce negative cancer outcomes, such as increase of biochemical recurrence (BCR) or cancer-specific mortality after RP^4^. Furthermore, some studies have presented poor longitudinal cancer control results among obese patients^5,6^. Despite various publications of study results supporting the association of obesity with PCa outcomes, several studies were unable to show body mass index (BMI) as a potential hazard of PCa, as they failed to detect profoundly poor outcomes of PCa in a cohort with metabolic syndrome and high BMI^7,8^.

For predicting adverse clinical outcomes after RP, prostate specific antigen (PSA), clinical stages and Gleason scores (GS) are the most frequently used predictors. In the present study, we hypothesized that BMI might be significantly associated with BCR and BCR-free survival as a negative predictor in PCa patients post-RP. Therefore, this study researched a cohort of RP patients to evaluate the relationship between BMI and BCR-free survival.

## Methods

### Study population and definitions

**Figure 1:**
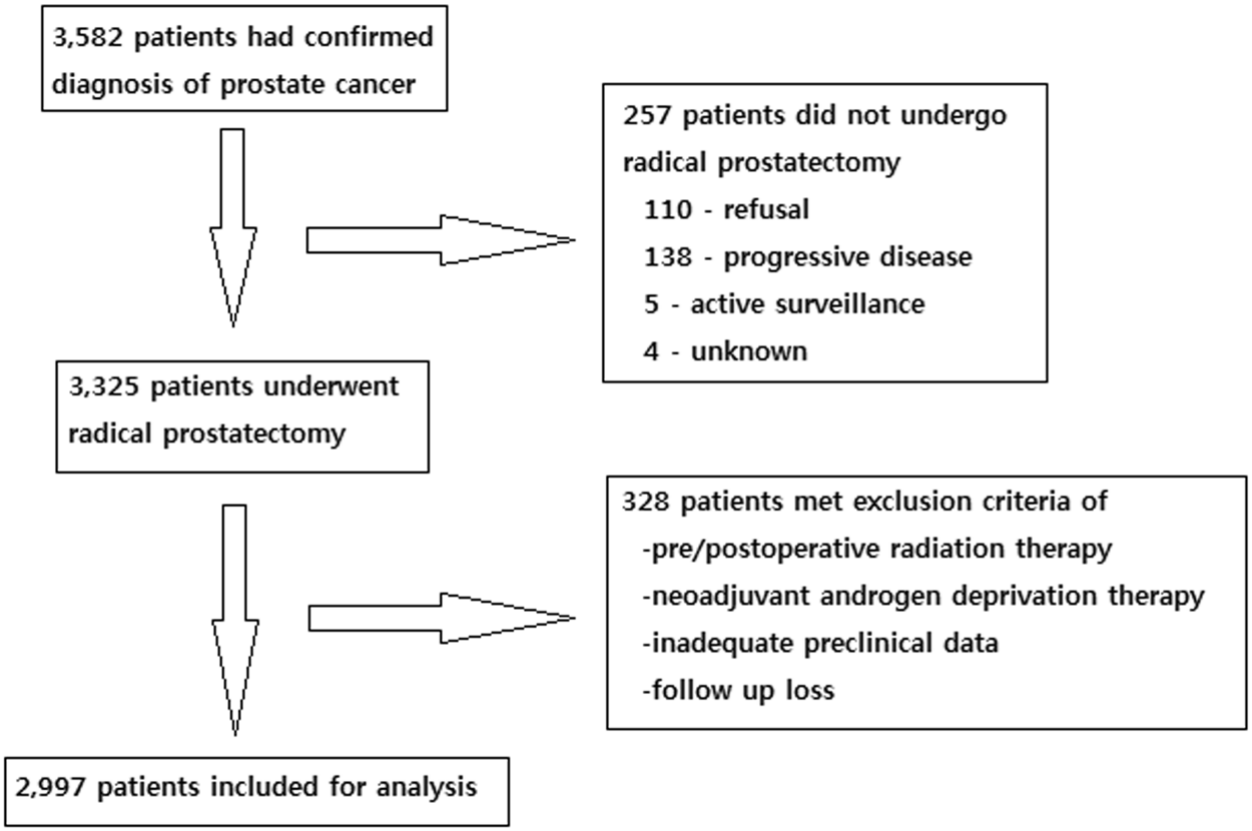
Patient allocation for the study.

After obtaining approval from the Seoul National University Bundang Hospital Institutional Review Board (IRB No. B-1712-439-103), written informed consent for medical research was obtained from all patients and their legal guardians before their inclusion in this research. All study procedures including data collection and management were performed in accordance with relevant guidelines and regulations. In turn, the medical records of patients with PCa were retrospectively reviewed. The patients included in this study underwent open radical prostatectomy (ORP), conventional laparoscopic radical prostatectomy (LRP) or robot-assisted laparoscopic radical prostatectomy (RALP) as their primary treatment for PCa at a single large-volume institution between January, 2006 and May, 2017. Four experienced staff surgeons performed all of the RP procedures without a preference for any specific surgical modality. Among the final study cohort, there were no patients who had received preoperative external beam radiation therapy (EBRT) or neoadjuvant androgen deprivation therapy (ADT). The patients with inadequate preclinical data or follow-up loss were excluded from the research design, which resulted in a total of 2,997 included patients out of 3,325 potentially eligible patients in the present study (Fig. 1). Magnetic resonance imaging (MRI) and bone scan were applied to all candidates during preoperative evaluation and no patient showed evidence of metastasis. Lymph node dissection was performed for the bilateral external and internal iliac vessels, and the obturator fossa according to the extension of lymph node involvement observed in the preoperative evaluation.

Age, BMI, year of RP, PSA, biopsy and pathological GS, clinical tumor stage (cT), pathological tumor stage (pT), margin positivity, surgical methods (ORP vs. LPR vs. RALP), extraprostatic invasion (EPI) status, continence recovery after RP, and potency were included as research variables. The present study used the continence definition of ≤1 pad per day, and the erectile function of the cohort was evaluated by using the International Index of Erectile Function (IIEF-5) questionnaire. The follow-up time (years) was calculated as the time form the date of prostatectomy to the last hospital visit. To evaluate the effect of BMI on PCa outcomes, BMI was stratified into three categories according to the World Health Organization (WHO) recommendations for Asian men as follows: normal weight (<23 kg/m^2^), overweight (≥23 to <27.5 kg/m^2^) and obese (≥27.5 kg/m^2^). According to the recent National Comprehensive Cancer Network (NCCN) guidelines^9^, the patients were also sorted into three preoperative risk groups as follows: low-risk = T1 to T2a, GS ≤ 6/Gleason grade group 1, and PSA < 10 ng/mL; intermediate-risk = T2b to T2c, or GS 3 + 4 = 7/Gleason grade group 2, or GS 4 + 3 = 7/Gleason grade group 3, or PSA 10–20 ng/mL; and high-risk = > T3a, or GS 8/Gleason grade group 4, or GS 9–10/Gleason grade group 5, or PSA > 20 ng/mL. BCR was defined as two consecutive postoperative PSA values ≥ 0.2 ng/ml.

### Statistical analysis

We analyzed the associations between clinical and pathological variables according to different BMI categories by using the Wilcoxon rank sum test. Chi-square tests and Student t-tests were performed to evaluate the differences between subgroups, including the patients with and without diabetes mellitus (DM). In multivariable logistic regression analysis, the effect of obesity and other clinicopathological variables on BCR rates and PCa specific survival (PCSM) were analyzed using unadjusted (crude) and adjusted competing risks. The adjusted data was balanced according to clinicopathological variables such as age, PSA, history of DM, pathological GS, pT, LNI, PSM, and surgical methods. Multivariable Cox regression analyses and Kaplan-Meier analyses were performed to test the effect of obesity on BCR-free survival. For continuous variables, means and interquartile ranges were reported and proportions were supplied for categorical variables. All statistical tests were performed using the SPSS package version 22.0 (SPSS Inc., Chicago, IL, USA), and *p* values < 0.05 were considered significant.

## Results

### Baseline characteristics

A total of 2,997 men with PCa who underwent RP were included in this study. The clinicopathological characteristics of the study subjects are presented in Table 1. Among the patients, normal weight (<23 kg/m^2^), overweight (≥23 to <27.5 kg/m^2^) and obese (≥27.5 kg/m^2^) men were 867 (29.9%), 1,799 (60.0%) and 331 (11.1%), respectively. The overall median follow-up time was 3.3 years, and no significant difference in follow-up time was observed between the BMI groups (*p* = 0.054). No significant preference of a specific surgical type was observed among the different BMI groups, and LRP was the least performed surgical method in all three BMI groups. The number of patients who underwent RALP, LRP, and ORP were 2,250 (75.1%), 23 (0.8%) and 724 (24.1%), respectively. The mean age of the patients was 66.0 ± 6.8 years with a range of 37–82 years and no significant differences were observed between the BMI groups. Other preoperative clinicopathological factors, including the mean baseline PSA level and biopsy GS, were similar among the BMI groups. Regarding pre- and postoperative clinicopathological factors, obese men showed higher rates of stage ≥cT3 (12.0%, *p* = 0.420) and pathologic GS ≥ 8 (50.8%, *p* = 0.017), although the percentage of patients with advanced cT was not significantly different among the groups. Moreover, obese patients had greater positive surgical margins (PSM) (13.9%, *p* < 0.001) and positive LNI rates (14.5%, *p* = 0.021) in their final pathological results than overweight and normal weight patients. The obese group also had a significantly higher percentage of patients confirmed with stage ≥ pT3b (17.8%, *p* = 0.002) compared to 13.8% of overweight and 7.0% of normal weight patients.

**Table 1:** Patient characteristics.

Parameter	Overall	BMI ≥ 27.5	BMI 23–27.5	BMI < 23	p-value
Patients, n (%)	2997	331 (11.1)	1799 (60.0)	867 (28.9)	
F/U time, year, mean (range)	3.3 (3.0–12.0)	3.1 (3.3–11.5)	3.5 (3.1–11.4)	3.6 (3.0–12.0)	**0.054**
Age, year, mean ± SD (range)	66.0 ± 6.8 (37–82)	65.3 ± 6.7 (51–76)	65.8 ± 7.1 (39–82)	66.1 ± 6.3 (40–80)	0.063
PSA, ng/dL, mean ± SD	12.9 ± 17.0	13.3 ± 17.1	12.8 ± 17.2	12.9 ± 16.8	0.596
DM, n (%)	462 (15.4)	86 (26.0)	304 (16.9)	72 (8.3)	<**0.001**
Biopsy, GS n (%)					**0.002**
≤6	1057 (35.3)	93 (28.0)	598 (33.2)	366 (42.2)	
7 (3 + 4)	964 (32.2)	85 (26.0)	592(32.9)	287 (33.1)	
7 (4 + 3)	436 (14.5)	61 (18.0)	261 (14.5)	114 (13.1)	
≥8	540 (18.0)	92 (28.0)	348 (19.4)	100 (11.6)	
Clinical T-stage, n (%)					0.420
cT1c	1823 (61.0)	199 (60.0)	1100 (61.0)	524 (60.0)	
cT2a	775 (25.9)	81 (24.5)	456 (25.3)	238 (27.5)	
cT2b	23 (0.8)	2 (0.6)	13 (0.7)	8 (0.9)	
cT2c	64 (2.1)	9 (2.7)	35 (1.9)	20 (2.3)	
≥cT3	312 (10.2)	40 (12.0)	195 (11.1)	77 (9.3)	
Pathological GS, n (%)					
≤6	55 (1.8)	5 (1.5)	5 (0.3)	45 (5.2)	**0.017**
7 (3 + 4)	905 (30.2)	81 (24.4)	540 (30.0)	284 (32.8)	
7 (4 + 3)	917 (30.6)	77 (23.3)	562 (31.2)	278 (32.0)	
≥8	1120 (37.4)	168 (50.8)	692 (38.5)	260 (30.0)	
Pathological T-stage, n (%)					**0.002**
pT2a	1154 (38.5)	73 (22.0)	689 (38.3)	392 (45.2)	
pT2b	933 (31.1)	128 (38.7)	540 (30.0)	265 (30.6)	
pT2c	370 (12.3)	34 (10.3)	207 (11.5)	129 (14.9)	
pT3a	172 (5.7)	37 (11.2)	115 (6.4)	20 (2.3)	
≥pT3b	368 (12.4)	59 (17.8)	248 (13.8)	61 (7.0)	
Extraprostatic invasion, n (%)	443 (14.8)	66 (19.9)	281 (15.6)	96 (11.1)	<**0.001**
Lymph node invasion, n (%)					**0.021**
Negative	2599 (86.7)	283 (85.5)	1541 (85.7)	775 (89.4)	
Positive	398 (13.3)	48 (14.5)	258 (14.3)	92 (10.6)	
Positive surgical margins, n (%)	105 (3.5)	46 (13.9)	47 (2.6)	12 (1.4)	<**0.001**
Surgical approach, n (%)					0.103
RALP	2250 (75.1)	250 (75.5)	1341 (74.5)	659 (76.0)	
LRP	23 (0.8)	2 (0.6)	15 (0.8)	6 (0.7)	
ORP	724 (24.1)	79 (23.9)	443 (24.7)	202 (23.3)	
BCR, n (%)	593 (19.8)	102 (30.9)	336 (18.6)	155 (17.8)	<**0.001**
BCR in RALP group	441/2250 (19.6)	75/250 (30.0)	246/1341 (18.3)	120/659 (18.2)	
BCR in LRP group	3/23(13.0)	2/2 (100.0)	1/15 (6.7)	0/6(0.0)	
BCR in ORP group	149/724 (20.5)	25/79 (31.6)	89/443 (20.1)	35/202 (17.3)	
Preoperative risk group					0.539
Low	1148 (38.3)	130 (39.3)	687 (38.2)	331 (38.2)	
Intermediate	718 (24.0)	79 (23.8)	432 (24)	208 (24)	
High	1131 (37.7)	122 (36.9)	680 (37.8)	328 (37.8)	
Early continence recovery after RP (≤3 months after RP), n (%)	2136 (71.3)	211 (63.7)	1240 (68.9)	685 (79.0)	<**0.001**
Total IIEF-5 score, mean ± SD					<**0.001**
Before RP	19.51 ± 7.87	18.93 ± 4.35	20.11 ± 5.12	21.45 ± 3.43	
6 months after RP	11.13 ± 5.63	8.49 ± 6.80	11.64 ± 7.28	13.22 ± 4.79	

F/U = follow-up; PSA = prostate specific antigen; DM = diabetes mellitus; GS = Gleason score; BCR = biochemical recurrence; RALP = robot-assisted laparoscopic prostatectomy; LRP = laparoscopic radical prostatectomy; ORP = open radical prostatectomy; BMI = body mass index; RP = radical prostatectomy; IIEF = international index of erectile function.

The EPI rates of the obese and overweight groups were 19.9% and 15.6%, respectively and these values were markedly higher than the 11.1% EPI rate of the normal weight group (*p* < 0.001). The obese group (BMI ≥ 27.5 kg/m^2^) had a significantly higher percentage of patients with DM (26%), compared to 16.9% and 8.3% of the patients in the overweight and normal weight groups, respectively (*p* < 0.001).

Among the study cohort, 593 patients (19.8%) experienced BCR during the follow-up period. The obese group showed profoundly higher BCR rates (30.9%) than the overweight (18.6%) and normal weight group (17.8%) (*p* < 0.001). In terms of surgical modalities, obese patients presented the highest BCR rates in all three surgical types (ORP 31.6%, LRP 100%, RALP 30.0%). Particularly, only two patients were categorized as obese among the 23LRP received patients, and both these patients underwent BCR.

In terms of continence recovery after RP, 2,136 patients among the study cohort (71.3%) had their continence recovery within ≤3 months from RP, which was defined as early continence recovery in the present study. Moreover, the obese group had a significantly lower rate of early continence recovery (63.7%) compared to the overweight (68.9%) and normal weight (79.0%) groups. In addition, the obese group showed more diminished baseline erectile function than the other two weight groups (total IIEF-5 score: obese = 18.93 ± 4.35 vs. overweight = 20.11 ± 5.12 vs. normal weight = 21.45 ± 3.43, *p* < 0.001). The total IIEF-5 score measured at 6 months after RP demonstrated that the obese group had the greatest average decrease in potency compared to the other BMI groups (obese = 8.49 ± 6.80 vs. overweight = 11.64 ± 7.28 vs. normal weight = 13.22 ± 4.79).

The results of subgroup analysis according to the presence of DM or age greater than 55 years are presented in Table 2. Diabetic patients had a significantly higher rate of obese patients (18.6%) compared to nondiabetic patients (9.6%) (*p* < 0.001). Moreover, diabetic patients showed significantly more adverse pathologic features compared with nondiabetic patients, as evidenced by the higher rates of pathological GS ≥ 8, stage ≥ pT3a, EPI, LNI, and PSM in diabetic patients (Table 2). Diabetic patients also showed a higher BCR rate than DM-negative patients (26.2% vs. 19.3%, *p* = 0.001). When evaluating the clinicopathological characteristics of patients by stratifying the cohorts into two subgroups, age > 55 and age ≤ 55, the younger group with age ≤55 had significantly higher proportions of poor oncologic factors including pathological GS ≥ 8, pathological stage ≥ pT3a, EPI, LNI, PSM, and BCR rates (Table 2).

**Table 2:** Differences in clinopathologic findings according to age and the presence of DM.

Parameter	DM positive	DM negative	p-value	Age > 55	Age ≤ 55	p-value
Patients, n	462	2535		2471	526	
Age, year, mean ± SD	66.2 ± 6.1	65.9 ± 6.8	0.102	68.4 ± 11.6	50.9 ± 4.1	<**0.001**
PSA, ng/dL, mean ± SD	12.9 ± 16.7	12.8 ± 17.2	0.378	13.2 ± 17.8	12.9 ± 18.1	0.053
HgbA1c ≥ 6.5%, n (%)	442 (95.7)	0 (0.0)	<**0.001**	647 (26.2)	133 (25.3)	0.120
BMI, kg/m^2^			<**0.001**			0.715
<23	72 (15.6)	795 (31.4)		566 (22.9)	123 (23.4)	
23–27.5	304 (65.8)	1495 (59.0)		1557 (63.0)	330 (62.7)	
≥27.5	86 (18.6)	245 (9.6)		348 (14.1)	73 (13.9)	
Pathological GS, n (%)			**0.002**			**0.036**
≤6	6 (1.3)	145 (5.7)		40 (1.6)	6 (1.1)	
7 (3 + 4)	72 (15.6)	1019 (40.2)		971 (39.3)	127 (24.2)	
7 (4 + 3)	147 (31.8)	796 (31.4)		655 (26.5)	164 (31.2)	
≥8	237 (51.3)	575 (22.7)		805 (32.6)	229 (43.5)	
Pathological T-stage, n (%)			<**0.001**			**0.002**
pT2a	105 (22.7)	1090 (43.0)		717 (29.0)	123 (23.4)	
pT2b	143 (31.0)	770 (30.4)		825 (33.4)	174 (33.0)	
pT2c	72 (15.6)	383 (15.1)		432 (17.5)	85 (16.2)	
pT3a	75 (16.2)	84 (3.3)		245 (9.9)	78 (14.8)	
≥pT3b	67 (14.5)	208 (8.2)		252 (10.2)	66 (12.5)	
Extraprostatic invasion, n (%)	98 (21.2)	274 (10.8)	<**0.001**	336 (13.6)	92 (17.5)	<**0.001**
LN invasion, n (%)	68 (14.7)	243 (9.6)	**0.010**	282 (11.4)	69 (13.1)	**0.021**
Positive surgical margins, n (%)	57 (13.4)	30 (1.2)	<**0.001**	178 (7.2)	62 (11.8)	**0.025**
BCR, n (%)	121 (26.2)	489 (19.3)	**0.001**	561 (22.7)	136 (25.9)	**0.039**

PSA: prostate specific antigen, HgbA1c: hemoglobin A1c, DM: diabetes mellitus, GS: Gleason score, RALP: robot-assisted laparoscopic prostatectomy, LRP: laparoscopic radical prostatectomy, ORP: open radical prostatectomy, BMI: body mass index.

### Multivariate analysis of BCR and PCSM

To evaluate the factors influencing BCR rates after RP, multivariate analyses were performed. Among the preoperative risk factors, PSA, DM, BMI, intermediate- and high-risk groups were significantly related to BCR (Table 3). In addition, pathological GS, pT, LNI, and PSM were independent predictors of BCR among the postoperative factors. In particular, all pathological stages that were more advanced than pT2a, achieved independent predictor status for BCR (Table 3). BMI ≥ 23 kg/m^2^ was also significantly associated with BCR in the multivariate analysis adjusted for clinicopathological variables including age, PSA, DM, pathological GS, pT, LNI, PSM and surgical methods (Table 3). In the multivariate analyses performed with unadjusted data, PCSM was significantly predicted by several factors such as BMI, intermediate- and high-preoperative risk groups, pT and LNI. Obesity with BMI ≥ 27.5 kg/m^2^ showed a significant association with PCSM, even in the multivariate analysis with post-adjustment data (Table 3).

**Table 3:** Multivariate and univariate analyses of the associations of clinicopathologic parameters with BCR and PCSM.

Parameter	BCR						PCSM		
	Univariate			Multivariate			Multivariate		
	OR	95% CI	p-value	OR	95% CI	p-value	OR	95% CI	p-value
Pre-operative factors (unadjusted)									
Age	0.171	0.166 to 0.173	0.896		—		1.083	0.766 to 1.253	0.718
PSA	1.106	1.102 to 1.117	0.047	3.740	1.071 to 15.011	<0.001	1.022	0.899 to 1.124	0.741
DM	1.184	1.010 to 1.762	0.011	1.036	1.011 to 2.373	0.003	0.865	0.754 to 1.545	0.930
BMI, kg/m^2^	1.147	1.022 to 1.875	0.007	2.058	1.013 to 4.102	0.014	1.015	1.006 to 1.340	0.025
Preoperative risk group									
Low	Reference			Reference			Reference		
Intermediate	3.260	1.829 to 4.561	<0.001	2.952	1.993–3.610	<0.001	3.239	2.196 to 4.331	0.012
High	4.228	2.905 to 4.970	<0.001	3.738	2.536–4.208	<0.001	4.204	3.001 to 5.872	<0.001
Postoperative factors (unadjusted)									
Pathological T-stage									
pT2a	Reference			Reference			Reference		
pT2b	5.227	3.005 to 9.012	0.008	1.875	1.118 to 5.632	0.006	1.821	1.247 to 2.103	<0.001
pT2c	2.548	2.109 to 3.004	0.023	3.841	3.713 to 3.968	<0.001	2.449	1.720 to 3.189	<0.001
pT3a	2.708	1.551 to 2.983	0.003	3.914	3.673 to 4.156	<0.001	2.843	1.507 to 3.715	<0.001
≥pT3b	4.792	1.120 to 12.513	<0.001	2.073	1.198 to 3.148	<0.001	3.307	1.890 to 5.578	<0.001
LNI	1.124	1.115 to 1.376	<0.001	2.537	2.310 to 2.764	<0.001	1.575	1.126 to 2.013	0.032
PSM	4.083	1.566 to 8.437	<0.001	3.205	2.017 to 5.457	<0.001	1.175	0.089 to 2.009	0.862
Surgical methods	0.087	0.071 to 0.102	0.207		—		1.210	0.852 to 1.648	0.981
Pathological, GS	3.169	1.853 to 10.784	<0.001	2.138	1.122 to 7.275	0.021	2.181	1.850 to 3.001	<0.001
Adjusted for clinicopathological factors (age, PSA, DM, pathological GS, pT, LNI, PSM, and surgical methods)									
BMI									
<23				Reference			Reference		
23–27.5				1.051	1.026 to 1.528	0.043	1.258	0.996 to 1.907	0.073
≥27.5				1.268	1.095 to 1.899	0.029	2.334	1.501 to 3.080	0.014

BCR = biochemical recurrence; PCSM = prostate cancer specific mortality; PSA = prostate specific antigen; DM = diabetes mellitus; BMI = body mass index; LNI = lymph node invasion; PSM = postive surgical margin; GS = Gleason score.

### Predictors of BCR-free survival

**Figure 2:**
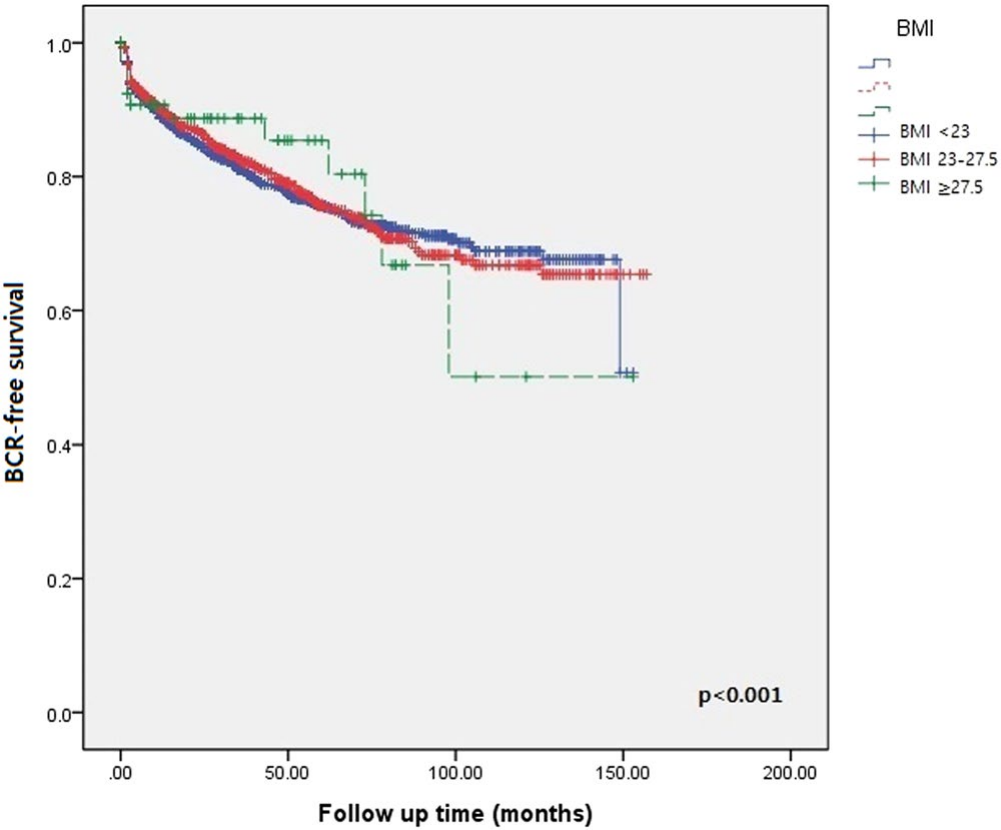
Kaplan-Meier curves for BCR-free survival of radical prostatectomy patients stratified by BMI; BCR = biochemical recurrence; BMI (kg/m^2^) = body mass index.

**Figure 3:**
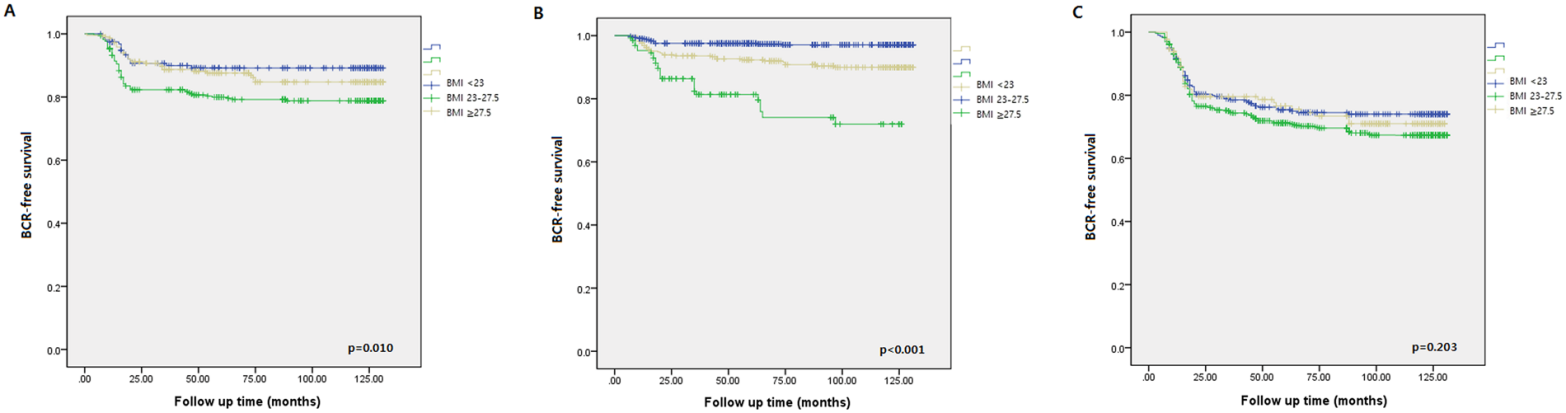
Kaplan-Meier curves for BCR-free survival stratified by patient BMI; A = National Comprehensive Cancer Network (NCCN) low-risk group; B = intermediate-risk group; C = high-risk group.

According to Kaplan-Meier analyses performed by sorting patients into three BMI categories, obesity was significantly correlated with BCR-free survival after RP, especially in the patients with BMI ≥ 27.5 kg/m^2^ (*p* < 0.001, Fig. 2). Upon categorizing the cohort into preoperative risk groups, low-risk and intermediate-risk patients (Fig. 3A,B) showed more significant differences in BCR-free survival between each BMI group, compared with the high-risk patients (Fig. 3C). Obesity with BMI ≥ 27.5 kg/m^2^ showed a significant impact on 5-year BCR-free survival among the low-risk group (75.6%), and the impact was greater for the intermediate- (81.4%) and high-risk groups (86.3%), as shown in Fig. 3.

According to multivariate Cox analyses, DM, LNI, pT, pathologic GS, EPI and PSM were statistically significant predictors of BCR-free survival after RP, but age and surgical methods were not (Table 4). Furthermore, obesity with BMI ≥ 27.5 kg/m^2^ was also an independent predictor of BCR-free survival (hazard ratio (HR) = 1.145; 95% confidence interval (CI): 1.109–1.183; *p* < 0.001; Table 3).

**Table 4:** Multivariate Cox analysis of BCR-free survival.

Parameter	BCR-free survival		
	HR	95% CI	p-value
Age	1.002	0.99 to 1.01	0.748
DM	1.162	1.070 to 1.852	**0.046**
Preoperative risk group			
Low	Reference		
Intermediate	2.128	1.482 to 3.523	<**0.001**
High	4.730	3.910 to 7.698	<**0.001**
Pathological T-stage			
pT2	Reference		
≥pT3	2.716	2.32 to 3.01	<**0.001**
Lymph node invasion			
pN0	Reference		
≥pN1	4.813	1.95 to 10.51	<**0.001**
BMI, kg/m^2^			
<23	Reference		
23–27.5	0.994	0.99 to 1.21	0.471
≥27.5	1.145	1.19 to 1.18	<**0.001**
Pathological GS			
≤6	Reference		
7 (3 + 4)	2.04	1.78 to 3.65	<**0.001**
7 (4 + 3)	2.95	2.14 to 3.92	<**0.001**
≥8	14.73	10.47 to 20.84	<**0.001**
Extraprostatic invasion			
Negative	Reference		
Positive	1.82	1.69 to 1.98	**0.031**
Surgical methods			
ORP	Reference		
LRP	1.02	0.75 to 1.09	0.863
RALP	1.28	0.97 to 1.80	0.720
Surgical margins			
NSM	Reference		
PSM	2.752	1.61 to 4.58	<**0.001**

PSA = prostate specific antigen; DM = diabetes mellitus; BMI = body mass index; GS = Gleason score; RALP = robot-assisted laparoscopic prostatectomy; LRP = laparoscopic radical prostatectomy; ORP = open radical prostatectomy; NSM = negative surgical margin; PSM = positive surgical margin.

## Discussion

Although many previous studies showed that obesity was associated with both aggressiveness of PCa at the time of diagnosis and decreased BCR-free survival^10–13^, the relationship between BMI and BCR is still debatable, as a significant number of prior studies failed to show an association between increased risk of BCR and higher BMI^14,15^. Thus, this study evaluated whether obesity (higher BMI) is a predictor of BCR and BCR-free survival after RP. The present study results presented that BMI was a significant independent predictor of poor outcomes in terms of BCR and BCR-free survival. Moreover, we found that comorbid DM was associated with BCR and BCR-free survival. Recent studies suggested that higher BMI was associated with an elevated risk of higher GS or higher cancer stage^16,17^, which coincides with our research results, in which the obese group (BMI ≥ 27.5 kg/m^2^) contained a significantly greater percentage of men with biopsy GS ≥ 8, stage ≥ cT3, stage ≥ pT3b and pathologic GS ≥ 8. Obese patients also showed a significant association with adverse surgical outcomes such as EPI, LNI and PSM in the current study. According to the results of the multivariate and Kaplan-Meier analyses, it could be postulated that obesity-associated pathological variables, such as higher GS, increased PSM and LNI, simultaneously influenced BCR-free survival to decrease in obese patients. Interestingly, a recent study showed that PCa patients primarily treated with EBRT had increases in BCR, metastases and cancer-specific mortality as BMI increased^11^. Since, no patients included in this study underwent neoadjuvant EBRT, the effect of radiation therapy on the cohort was minimal from the beginning of the analysis.

There are several explanations for poor oncologic outcomes in obese patients, and technical difficulties of performing surgery on obese men may be one of them. Previous studies have suggested that obesity increases the chance of capsular incision by 30%, and hinders the ideal performance of RP^18^. Other surgical challenges, such as more difficult lymph node dissection or nerve sparing, might also induce poor outcomes in oncological features. Furthermore, as BCR and BCR-free survival after RP were similar among the different surgical methods in this study, no technical inferiority of a specific surgical method was observed within the cohort. We believe this might be a result of the technical maturity of the surgeons who participated in the present study. Another possible explanation for the association of obesity with poor oncologic outcomes focuses on biological factors. First, obese patients show increased serum insulin, insulin like growth factor-1, interleukin-6 and leptin levels^19,20^. In contrast, some studies showed decreased adiponectin and serum testosterone levels in obese males^21–23^. Moreover, a retrospective study by Ma and colleagues analyzed the relationship between cancer-specific survival and C-peptide level, which directly represents insulin level, in PCa patients^5^. According to their research, the effect of BMI on PCa-specific survival was partly controlled by C-peptide level. However, the validity of these factors still needs to be evaluated in further studies.

In this study, the mean age of the patients was similar between BMI groups. However, the obese group showed a 26.0% DM prevalence, which was significantly higher than the other BMI groups. These outcomes are similar to the results of a previous epidemiological study that showed middle to old age men (age > 54 years) had a greater prevalence of DM in the obese or overweight population^24^. In addition, a prior study reported that the increased prevalence of DM diminished the adverse effects of obesity on BCR in PCa patients^7^. However, our findings showed diabetic patients had higher BCR rates than nondiabetic patients and 95.7% of the DM positive patients had hemoglobin A1c (HgbA1c) ≥ 6.5%. In our previous study published in 2015^25^, we showed that poor glycemic control with HgbA1c ≥ 6.5% was negatively associated with BCR rates after RP. Therefore, a relatively high proportion of patients with inadequate glycemic control in DM-positive group might have caused poor prognosis in terms of BCR. Ji *et al*. recently reported that PCa patients with age ≤55 or >75 years are likely to have more aggressive disease compared to the patients between the ages of 55 and 75. In this study, we observed similar results as the patients aged ≤55 years had more adverse pathologic features, including stage ≥ pT3b and higher BCR rate than the patients > 55 years old. Howeverm further studies are needed to confirm the association between specific age groups and PCa aggressiveness.

Yamoah *et al*.^26^ reported in their study that the intermediate- and high-risk groups had a worse 7-year freedom from biochemical failure among obese patients (BMI ≥ 30 kg/m^2^) compared with the low-risk group. This study showed similar results by presenting 75.6% 5-year BCR-free survival in the high-risk group among obese patients (BMI ≥ 27.5 kg/m^2^), whereas the intermediate- and low-risk groups showed 86.3% and 81.4%, respectively. However, in terms of overall BCR-free survival, the high-risk group had a smaller difference between each BMI group with no statistical significance (p = 0.203). According to the corresponding study results, we believe obese patients with NCCN high-risk should be more carefully approached than normal weight patients during the planning of appropriate treatment option, to provide the best oncological outcomes.

A prior meta-analysis study described that an increase of BMI by 5 kg/m^2^ caused the risk of PCSM to be increased by 20%^15^. Another previous study presented that higher BMI was associated with greater PCSM in RP patients (HR 1.58 for BMI ≥ 35 kg/m^2^)^27^. Our results also suggested obesity with higher BMI might be a risk factor for PCSM and the findings were similar to the prior research outcomes. However, as either more aggressive PCa or less aggressive treatment can cause a variation of PCSM^25^, further evaluations that account for the effect of adjuvant therapy are required to confirm the association between higher BMI and PCSM.

There have been few studies that evaluated the association of obesity with poor oncologic outcomes of PCa in the Asian population. As this study assessed a purely Asian population with PCa, the difference between our study results and other studies focused on Western ethnic groups might be the result of variances in obesity prevalence.

For further evaluation, we also conducted additional analyses regarding the relationship between BMI and continence recovery after RP. According to our results, obese men had a significantly lower rate of early continence recovery after RP compared to normal or overweight patients. This result is similar to the previous studies^28,29^, including the study of Bacon *et al*.^28^, which presented normal weight patients having a significantly higher continence recovery rates than overweight and obese patients at 12 months post RP (continence rate: 70% vs 68% vs 57%, *p* = 0.03). Due to the corresponding results concerning continence recovery, we believe that obese patients should be consulted for the higher risk of post-RP at the begging of surgical planning. Moreover, anti-incontinence surgical techniques including bladder neck preservation should be actively considered when RP is performed.

Some previous studies suggested an association between obesity and erectile function^29,30^. In a study of Wiltz *et al*.^29^, they showed that obese patients had poorer preoperative potency than normal weight and overweight males. Ahlering *et al*.^31^ also presented that obese patients had persistently lower potency rates compared with other weight groups during 24 months follow-up after RALP. Our study results showed that both pre- and post-RP erectile function was poorer in obese patients, and these results are similar to the outcomes from the previous results^30,31^. We believe higher risks of endothelial dysfunction and a greater rates of medical comorbidities, including DM caused profound erectile dysfunction in obese patients.

This study had several limitations. It was a retrospective cross-sectional study based on the medical records from a single institution. Thus, the treatments of the study cohort, such as postoperative ADT type and duration, and RT protocols, were heterogeneous, and a further research is needed to confirm whether the results are applicable to the general population or other ethnic groups. We used only BMI to represent obesity. BMI may not be useful in distinguishing pure adipose tissue from lean body mass in patients with relatively larger proportions of muscle mass because BMI encompasses lean body mass in its definition. Thus, other obesity indicators such as the amount of visceral adipose tissue, which is often represented by waist circumference, need to be included in further studies. Moreover, further studies with longer follow-up periods and molecular-level evaluation of the effects of obesity on oncological outcomes are needed.

## Conclusions

In conclusion, this study analyzed the effects of obesity (higher BMI) on BCR and BCR-free survival after RP in Asian population with PCa. Our study results demonstrated that obesity was significantly associated with BCR in PCa patients post-RP. Obesity (BMI ≥ 27.5 kg/m^2^) was a strong independent predictor of BCR-free survival. However, confirmation of the obesity influence on PCa remains debatable, and further studies, including consideration of pathophysiologic mechanisms at the molecular-level, are warranted.
